# A Preparative Mass Spectrometer to Deposit Intact Large Native Protein Complexes

**DOI:** 10.1021/acsnano.2c04831

**Published:** 2022-08-29

**Authors:** Paul Fremdling, Tim K. Esser, Bodhisattwa Saha, Alexander A. Makarov, Kyle L. Fort, Maria Reinhardt-Szyba, Joseph Gault, Stephan Rauschenbach

**Affiliations:** † Chemistry Research Laboratory, Department of Chemistry, University of Oxford, 12 Mansfield Road, Oxford OX1 3TA, United Kingdom; ‡ Thermo Fisher Scientific, Bremen 28199, Germany; § Biomolecular Mass Spectrometry and Proteomics, Bijvoet Center for Biomolecular Research and Utrecht Institute for Pharmaceutical Sciences, University of Utrecht, Padualaan 8, 3584 CH Utrecht, The Netherlands; ∥ Max Planck Institute for Solid State Research, Heisenbergstrasse 1, Stuttgart 70569, Germany

**Keywords:** soft landing, ES-IBD, native MS, enzymatic activity, TEM, transmission, energy width

## Abstract

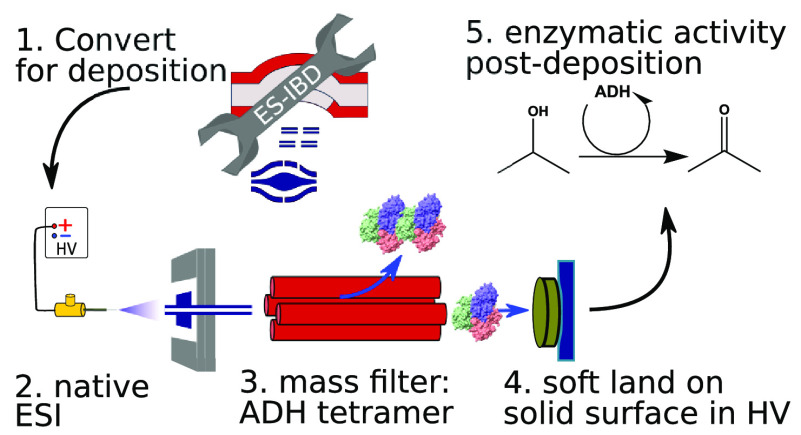

Electrospray ion-beam deposition (ES-IBD) is a versatile tool to study the structure and reactivity of molecules from small metal clusters to large protein assemblies. It brings molecules gently into the gas phase, where they can be accurately manipulated and purified, followed by controlled deposition onto various substrates. In combination with imaging techniques, direct structural information on well-defined molecules can be obtained, which is essential to test and interpret results from indirect mass spectrometry techniques. To date, ion-beam deposition experiments are limited to a small number of custom instruments worldwide, and there are no commercial alternatives. Here we present a module that adds ion-beam deposition capabilities to a popular commercial MS platform (Thermo Scientific Q Exactive UHMR mass spectrometer). This combination significantly reduces the overhead associated with custom instruments, while benefiting from established high performance and reliability. We present current performance characteristics including beam intensity, landing-energy control, and deposition spot size for a broad range of molecules. In combination with atomic force microscopy (AFM) and transmission electron microscopy (TEM), we distinguish near-native from unfolded proteins and show retention of the native shape of protein assemblies after dehydration and deposition. Further, we use an enzymatic assay to quantify the activity of a noncovalent protein complex after deposition on a dry surface. Together, these results not only indicate a great potential of ES-IBD for applications in structural biology, but also outline the challenges that need to be solved for it to reach its full potential.

Cryogenic electron microscopy (cryo-EM), low-energy electron holography (LEEH), and scanning probe microscopy (SPM) are complementary imaging techniques to probe the structure and conformation of biomolecules at sub-nanometer resolution.^1−5^ Cryo-EM has evolved into a leading method for high-resolution imaging of biological macromolecules.^6−8^ LEEH is a low-energy electron, single-particle microscopy method that allows imaging of highly flexible proteins in their individual conformations.^9^ SPM reveals the connectivity of branched oligosaccharides^5^ and allows access to the electronic structure of individual molecules.^10,11^ All three methods require samples produced at the highest standard to work optimally. LEEH and high-resolution SPM require ultrapure, UHV-compatible substrate conditions and greatly profit from chemical purity of the adsorbate.^2^ For cryo-EM, the preparation of homogeneous, high-quality samples can be challenging, especially for complex biomolecules. Conventional sample preparation for cryo-EM proceeds through the plunge freezing method, which has been enormously successful, but can be time-consuming and resource-intensive, and homogeneity is limited by solution-based purification techniques.^12−15^

Electrospray ion beam deposition (ES-IBD) is a preparative mass spectrometry^16,17^ technique, capable of producing highly purified molecular samples for single molecule imaging. It is routinely used for SPM with smaller (bio)molecules^5,18−24^ and has been demonstrated also for TEM,^25−28^ LEEH,^2,9^ and recently cryo-EM.^29,30^ In contrast to organic molecular beam epitaxy (OMBE),^31,32^ ES-IBD is not limited to small and volatile molecules. In ES-IBD, molecules are ionized in an electrospray ion source, transferred into the gas phase, and mass-analyzed in a vacuum. Then, the ion beam is mass-to-charge-ratio filtered and deposited with a controlled landing energy onto a suitable substrate. ES-IBD is often referred to as “soft landing” at lower collision energies, or “reactive landing” at higher collision energies or if the collision results in formation of a covalent bond to the surface. It enables reaction pathways inaccessible with other techniques^33,34^ and surface modifications.^35^

In addition to the requirements for ESI mass spectrometry, ES-IBD needs an intense ion beam^18,36−38^ with well-defined energy distribution to enable fast sample preparation with controlled landing energy. The width of the beam-energy distribution is crucial, as it defines the collision energy distribution and limits the landing energy range. Reported values for full width at half-maximum (FWHM) range from 2 to 10 eV per charge.^18,33,39,40^ The beam-energy width determines the minimal landing energy, which can be used without deflecting a significant portion of the ion beam. Narrow beam-energy distributions enable controlled exploration of shallow conformation spaces.^41^ A low landing energy per charge is important for highly charged protein complexes, as their absolute landing energy is proportional to the charge state. For example, the heavy native protein complex ion GroEL^+67^ experiences 670 eV absolute landing energy at a landing energy set to 10 eV per charge.

Likewise, the beam intensity determines the deposition time for a given deposition area and particle density. We use the charge of the deposited ions to quantify the deposited amount, given in the unit pAh (1 pAh = 3.6 × 10^–9^ C = 2.2 × 10^10^ e). In practice, 5–20 pAh are sufficient for imaging.^2,9,24,29^ This charge allows depositing on a several mm^2^ large sample with a sub-monolayer coverage that enables imaging of isolated particles. Beam currents of more than 20 pA ensure typical deposition times of less than half an hour, so multiple deposition conditions can be tested in a day. However, precise mass-selection inherently reduces the current available for deposition, since all ions except the selected one are removed from the beam. Finally, an accurate current measurement on the level of 1 pA is needed to achieve reproducible coverage.

For sample preparation of biological macromolecules, the structural integrity of fragile biomolecules has to be maintained for the entire ES-IBD process. Native MS retains covalent and most noncovalent interactions within a protein complex^42−44^ and can be integrated to ES-IBD. Nevertheless, it remains unclear to which extent ionization, liquid–gas-phase-vacuum transfer, and soft landing affect noncovalent interactions and hence the conformation and structure of the protein complexes.

Currently, the barrier to widespread use of ES-IBD is still high, and there is no commercial instrument available. Academic instrument developers have designed preparative MS mainly for small and medium size molecule deposition,^20,37,40,45−50^ and only a few of these instruments can handle native protein complexes.^2,9,28^ To be universally useful for molecular ion-beam deposition, ES-IBD instruments need to be good mass spectrometers, and have a high beam current in addition to the features needed for beam control and deposition.

Commercial, analytical mass spectrometers combine high-resolution mass analyzers^51^ with a user interface focused on mass spectrometry experiments. However, they have insufficient beam intensity for ES-IBD and lack the flexibility in design and software to integrate deposition as an additional workflow. As a minimum requirement, a native ES-IBD/MS must handle large, low-mass-to-charge-ratio protein ions with a molecular weight of up to a megadalton. While some home-built or converted machines can do this,^2,9,30,39,52^ their mass filter, collisional activation, or beam control is severely restricted in comparison to commercial instruments.

Here, we show how to convert a proven, commercial, analytical, native mass spectrometer to a native ES-IBD platform. It has an intense, well-controlled ion beam, which we characterize with current and energy measurements. Three different methods are used to demonstrate that the platform is suitable for near-native deposition: Protein heights observed in SPM images show globular features when preparing samples using native ES-IBD, compared to denatured, conventional MS conditions. Using TEM, we demonstrate the importance of landing energy control to preserve near-native structural features. Finally, we show that a noncovalent enzymatic complex retains activity after ES-IBD.

## Results and Discussion

### Instrument Setup and Modification

**Figure 1:**
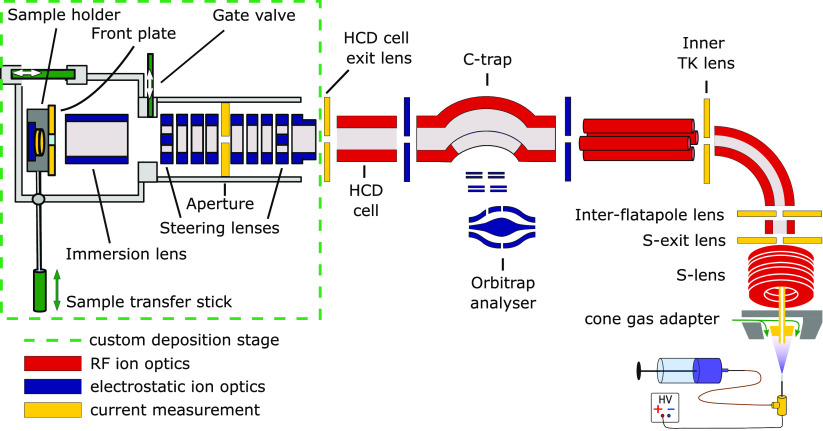
Schematic view of the Q Exactive UHMR mass spectrometer modified for deposition. Custom landing stage to deposit two microscopy samples and measure energy on the left. UHMR with improved source for better transmission on the right.

We have converted a Q Exactive UHMR instrument (Thermo Fisher Scientific, Bremen, Germany) into a preparative mass spectrometer by adding a custom-built landing stage downstream of the higher energy collisional dissociation (HCD) cell. Figure 1 shows a scheme of the instrument. The added stage contains electrostatic lenses to focus and steer the beam onto a sample holder, containing two sample positions and a retarding grid energy detector (this scheme only shows a single sample in the sample holder).

A sample transfer rod moves the samples in and out of the deposition chamber. That process takes 2 min including pumping and venting. To monitor the beam intensity, the ion current is measured at the landing stage and on apertures throughout the instrument, which were modified to add this capability (yellow elements in Figure 1). In addition, we have increased the S-exit lens diameter from 1.4 to 2.5 mm and added a custom cone gas adapter to increase transmission efficiency and thus achieve shorter deposition times (see Methods).

### Deposition Workflow

First, we load up to two samples, typically TEM grids or highly oriented pyrolytic graphite (HOPG) substrates, into the sample holder and insert them into the deposition stage. We create an ion beam and check the composition with the Orbitrap mass analyzer and set the quadrupole mass filter to select the species required for deposition.

To optimize the beam intensity for deposition, we switch to beam mode. In this mode, the C-trap and the HCD cell guide the ions in a continuous beam, instead of intermittently pulsing the beam into the Orbitrap mass analyzer. All direct current (DC) potentials within the Q Exactive UHMR instrument were kept at default values, which minimize activation during transmission from source to the deposition stage (see Figure S1a). This usually means that potential gradients are as low as possible especially in regions where collisions with the background gas occur.

Next, the beam is steered onto the energy detector. In front of the collector plate that is used to measure the ion current, the detector has a metal grid to apply retarding voltages. Ions with a total energy below their potential energy at the grid cannot reach the detector plate. Hence, we record the ion current at the detector plate as a function of the grid potential to obtain the beam energy.

The difference between the beam energy and the retarding sample potential determines the landing energy. We typically use a range from 2 to 100 eV per charge depending on the specific application. For deposition, we finally steer the beam onto the sample and start integrating the detected sample current, to measure when the desired coverage is achieved. During deposition the beam composition is checked periodically using the mass analyzer.

### Beam-Energy Distribution

The total energy of the ion beam, its distribution, and the sample potential define the collision energy with the surface. The total energy distribution is determined by the potential along the beam path and the interactions of the ions with the background gas. Hence, it can be influenced by the local pressure in the ion optics and by the applied radio frequency (RF) and DC voltages. The pressure ranges from 0.01 mbar in the HCD cell to high vacuum in the landing stage.

In our instrument, total energy is measured via the retarding grid detector integrated in the sample holder (see Figure 1 and Methods). We investigated the influence of two distinct sets of potentials on the beam-energy distribution, one with higher and one with lower potential gradients in the landing stage (see Figure S1b).

For this investigation, we used an ion-beam of denatured and a native bovine serum albumin (BSA). Denatured BSA yields a wide range of charge states between +44 and +15 (1600–4500 Th, Figure S3a, 1 Th = 1 Da · *e*^–1^). The native BSA beam contains the monomer as well as undefined, higher-order aggregates. Their mass-to-charge ratio is 3900 (+17, monomer) to 10 200 Th (aggregate, Figure S3b). The detector measures the beam’s intensity and the total energy (*E*_tot_). *E*_tot_ is the sum of the ion’s kinetic *E*_kin_ and potential energy *E*_pot_. *E*_kin_ only depends on the ion’s velocity. Its *E*_pot_ depends on charge state and position in the electric potential landscape. The reference for *E*_pot_ and *E*_tot_ is electrical ground. Hence, an ion with a negative *E*_tot_ cannot reach a grounded electrode.

**Figure 2:**
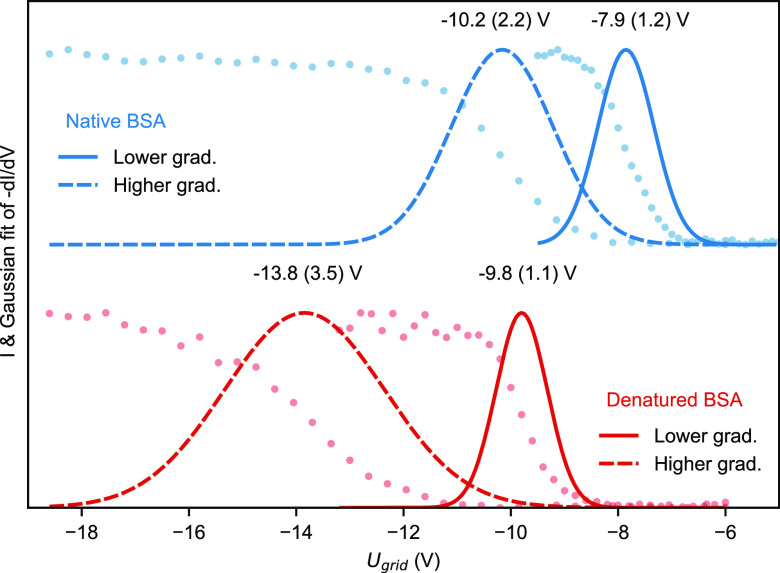
Beam-energy distribution measured for denatured and native BSA ion beams for two different potential gradient settings as shown in Figure S1b. Dots: Ion current measured as a function of the retarding grid potential *I*(*U*_bias_). Lines: Gaussian fit for first derivative d*I*/d*U*_bias_, corresponding to the total beam energy per charge (*E*_tot_) in eV per charge. FWHM is given in parentheses.

Figure 2 shows beam-energy distributions measured under different conditions. They are represented as Gaussian fits to the first derivative of the beam current, *I*, with respect to the grid bias, *U*_grid_. The grid potential *U*_grid_ corresponds to *E*_tot_. All measured *E*_tot_ are negative. Consequently, all ion optics in the landing stage have to shield the ion beam path from the potential of the grounded vacuum chamber. To this end, the ion optics fully enclose the beam path and have a negative potential applied.

The state of the ion, folded or unfolded, as well as the chosen potential landscape influence the *E*_tot_ mean value and *E*_tot_ distribution width, in the following given as *E*(Δ*E*). When a lower DC gradient for focusing within the electrostatic lens was applied, the denatured BSA *E*_tot_ was −9.8(1.1) eV per charge. It is lower by 4 eV per charge and widens by 2.4 eV per charge when choosing a higher gradient instead. Native BSA’s *E*_tot_ follows a similar trend, albeit with a higher *E*_tot_ mean of −7.9 eV per charge with the lower gradient and −10.2 eV per charge for the higher gradient.

The interplay between local pressure and ion acceleration in the electrostatic lens determines *E*_tot_. Ions thermalize in the HCD cell to an *E*_tot_ of −5 eV per charge, which is defined by the axial DC-potential. From there they enter the electrostatic lenses. Although the pressure rapidly decreases, the ion’s mean free path is significantly shorter than the distance between the HCD exit lens and the next aperture and hence energetic ion-background gas collisions will occur. The ions gain kinetic energy (*E*_kin_) between two collisions proportional to the DC gradient (electric field, see Figure S1b) along the flight path in the landing stage. The relative loss of kinetic energy per collision depends mainly on the mass of the collision partners, with the absolute loss per collision higher at higher *E*_kin_. The randomness of the impact angle between gas and ion causes a distribution in energy loss, which is wider for high *E*_kin_. Thus, a high potential gradient causes a large decrease in *E*_tot_ and widens Δ*E*_tot_, the width of the distribution (see the SI).

Two factors explain the lower *E*_tot_ for the denatured protein. First, the number of collisions in the electrostatic lens increases with the unfolded protein’s larger collisional cross section.^53^ Second, the denatured protein ions’ higher charge states raise the overall *E*_kin_ (for the same value of energy per charge), which leads to higher energy loss in collisions as compared to the low charge state, native ion.

In summary, when transferring an ion beam from high-pressure RF optics into high vacuum, the magnitude and distribution of *E*_tot_ are a function of the DC gradient, background pressure, ion charge, and collision cross section (CCS). For a given type of ion, efficient pumping and a weak DC gradient ensure a narrow distribution of total beam energy, enabling all ions to land on a substrate downstream with a similar collision energy.

Here, using low gradients, the *E*_tot_ distribution (FWHM  per charge) is sharper than previously reported literature values (FWHM  per charge),^18,21,33,40^ pointing to gentle conditions in which gas-phase activation is minimal. Given that the lower gradient conditions also achieved high transmission and good beam focus, we retained them for all other experiments presented here.

### Transmission

**Figure 3:**
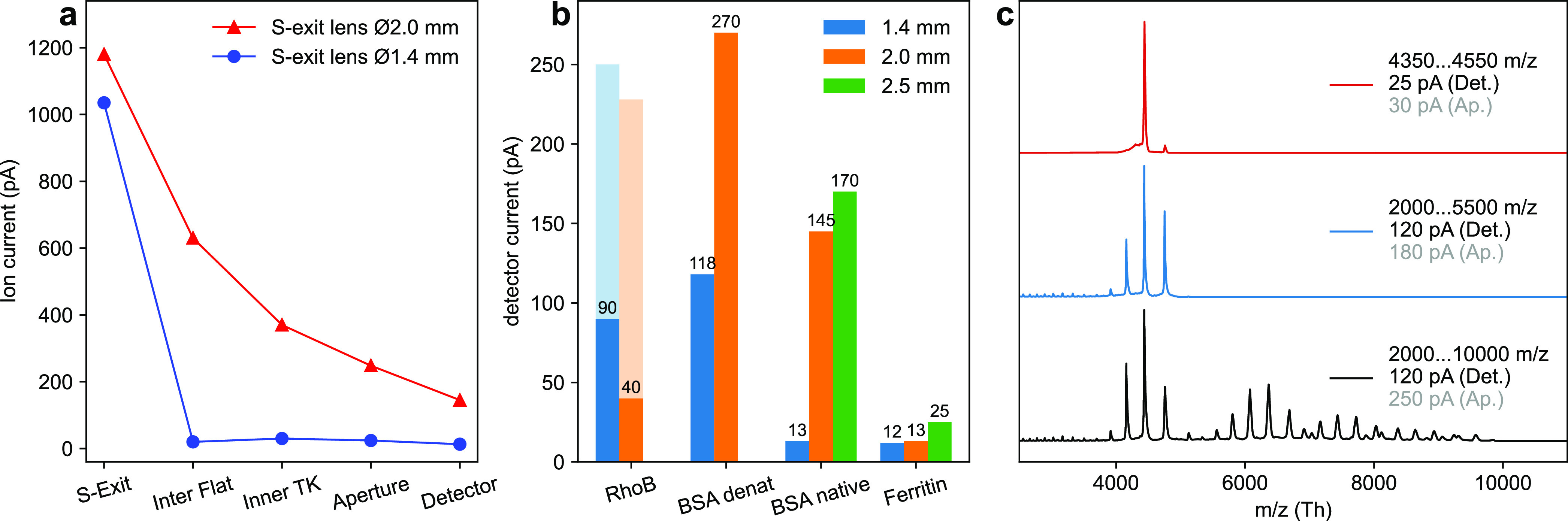
Transmission properties. (a) Ion current across the instrument before and after increasing the S-exit-lens diameter, measured at different ion optics. (b) Typical ion currents at the energy detector for different S-lens diameters. (Values equivalent to sample currents). Protein ion currents increase with aperture size. RhoB currents do not follow the trend, due to a defocusing effect. Currents on preceding optical elements are shown in light colors. (c) Native BSA currents on energy detector (Det.) and aperture (Ap.) decrease with the narrowing width of the mass filter window.

High transmission is crucial for deposition experiments, since the particle flux directly determines the deposition time for a given coverage and sample surface area. Using a typical analyte concentration of 3 μmol L^–1^ and assuming a 1 μL h^–1^ nanoelectrospray flow rate with 100% ionization efficiency, a 1.2 nA emission current of native BSA (*z* = 15) would be generated. This emission current is the upper limit estimation for the possible current of a 3 μmol L^–1^ BSA solution (see SI for details). However, under these conditions we measured initially only 13 pA at the sample position in the Q Exactive UHMR instrument with an unmodified source region. An initial measurement indicated a 1 nA current in the first vacuum chamber (Figure 3a). This may include ionized solvent and contaminants. There was also a sharp drop in current between the S exit lens and the inter-flatapole lens.

To improve the transmission performance, we enlarged the inner diameter of the S-exit lens stepwise from 1.4 to 2.0 and finally to 2.5 mm. With the 2.5 mm opening, the ion current at the sample for large, native proteins doubled to 25 pA and for medium sized, native proteins the current grew more than 10-fold to 170 pA (Figure 3b). There was no measurable effect for Rhodamine B (RhoB), a relatively small ion with an *m*/*z* of 443 Th. All currents reported here are routinely reached with fluctuations of up to 80%, due to emitter performance.

The overall transmission is further affected by mass-filtering, where a narrow *m*/*z*-window not only suppresses contamination, but can also reduce the flux of desired analyte molecules. Figure 3c illustrates how the width of the mass-filter window affects the native BSA current: Removing higher-order agglomerates has no effect on the sample current (bottom to mid-panel). Under the used conditions, the higher-order agglomerates are in the fringe of the ion beam, so they hit the sample holder front plate instead of the sample. It was possible to filter a single charge state while retaining a third of the total current.

In contrast to the protein ion currents, RhoB current does not change with increasing S-lens diameters. Likely, a different beam profile as compared to heavy protein ions causes this behavior. Thanks to its low *m*/*z*, RhoB experiences a stronger effective potential than high *m*/*z* protein ions within the S-lens. Thus, it can remain closer to the optical axis, reducing losses at the transfer apertures.

The modifications to increase the ion current are vital for depositing larger molecules. They allow testing of several deposition conditions on a single experiment day, where particle densities of 3000 μm^–2^ or more are needed for efficient cryo-EM or SPM. For our applications, this is usually achieved with a deposited charge of 15 pAh. The modifications lead to a deposition time of approximately 0.5 h for large native protein complexes.

While necessary for preparative MS, our modifications cause the gas flow into the injection flatapole collision cell to become significantly higher. The pressure in the flatapole rises as a consequence and could decrease the in-source-trapping effectiveness.

### Ion-Beam Shape and Control

The ability to create a narrowly focused beam is essential to reduce the time needed to achieve the optimal particle density for SPM or TEM. We used three different methods to assess the ion beam profile under typical experimental conditions.

**Figure 4:**
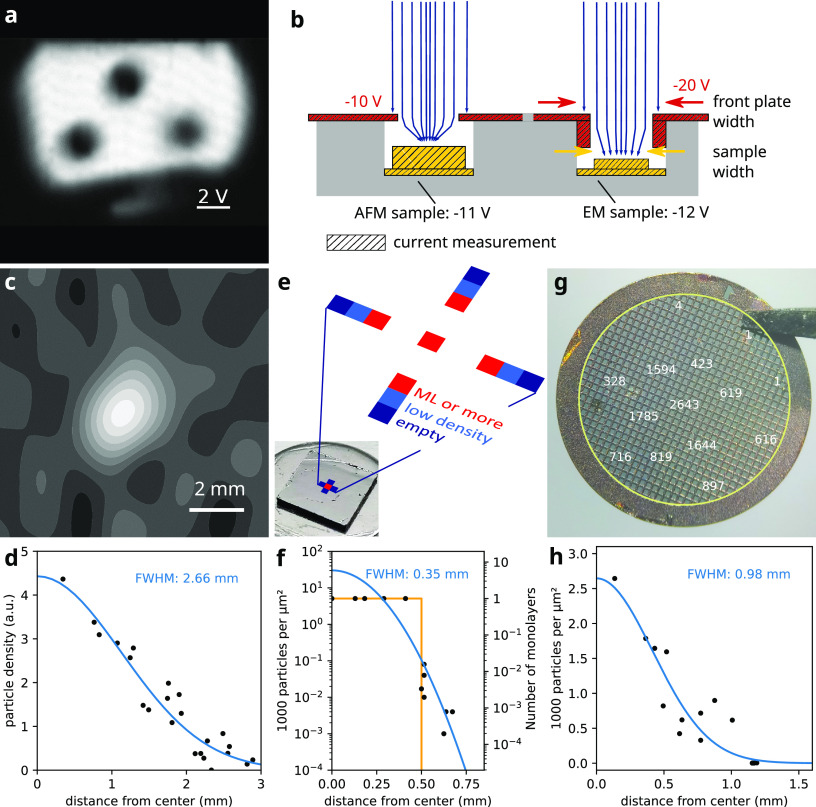
Ion beam shape analysis. (a) Ion-beam image of the sample holder front plate. (b) Sample holder section view: Different voltages influence focusing. (c) Ion-beam shape from deconvolution of (a). (d) Data points and Gaussian fit of ion-beam intensity distribution. (e) HOPG sample used for AFM measurements and protein density distribution in the screened area. (f) Measured protein distribution on (e) (black dots), Gaussian fit (blue), and single monolayer model (orange). (g) Amorphous carbon grid used for TEM measurements and protein density distribution in particles per μm^2^. (h) Gaussian fit (blue) of density distribution in (g). Individual AFM and TEM micrographs are shown in Figure S4 in the SI.

First, we took an ion-beam image of the front plate of our sample holder (see Figure 4a and Figure S14). For this, we scanned the beam with the deflection elements in the electrostatic lenses and recorded the current on the front plate. The resulting current image is a convolution of the front plate geometry and the beam shape. Deconvolution revealed a Gaussian-like beam profile (shown in Figure 4c). A Gaussian fit gives a FWHM of 2.7 mm, only slightly larger than the diameter of the preceding aperture of 2 mm, which the beam typically passes without losses. The observed widening between the last aperture and the front plate is a consequence of the beam-energy distribution and the DC gradient in this section. A weak DC gradient moves the ions slowly in axial direction and gives them more time to expand radially. The beam profile obtained in this way is the profile at the front plate, whereas the samples are located a few mm behind and can be biased at a different potential.

The beam profile is different on the sample, because the potential gradient between front plate and sample can focus the beam (Figure 4b). We used AFM to determine protein density distribution after ion beam deposition on HOPG and TEM imaging after deposition on a TEM grid.

We typically use 5 mm wide HOPG chips (see Figure 4e) as substrates for AFM imaging. For the example given here, we deposited 12.5 pAh of GroEL. Multiple AFM images were taken on the graphite sample, distributed along the length and width of the sample. We found that, for the specific DC potentials used in this experiment, most of the surface area was empty and proteins were localized in a small spot near the center. Surprisingly, we observed a transition from a clean, empty surface to a coverage of more than a monolayer within 250 μm. We estimate the total number of GroEL particles as 4.2 × 10^9^ , from the deposited charge and average charge state of +67.

Because AFM cannot distinguish between single and multiple monolayer coverage, we can only roughly approximate the particle distribution. We fitted our data to two alternative models. A Gaussian fit combines the total particle number with the particle density in the sub-monolayer coverage area. It suggests a deposition spot FWHM of just 350 μm and a coverage of up to six monolayers at the center. However, it fails to reproduce the sharp increase in density at the spot’s boundary. Alternatively, we assume a monolayer density in the center (ca. 5000 particles per μm^2^) with a sharp drop to 0 at 0.5 mm from the spot center (orange curve in Figure 4f). This model overestimates the density at the spot boundary. The real distribution is likely found between these two estimates. As changing position on the sample can be tedious in AFM, other methods with wider field of view or faster change of position would be more appropriate to analyze particle distributions.

Thus, as a third approach, we deposited an apo/holoferritin mixture on a TEM grid covered with 3 nm amorphous carbon film (see Figure 4g) and acquired micrographs at room temperature. The density of holoferritin iron cores was quantified on different grid squares. The resulting distribution is shown in Figure 4h, together with Gaussian fit. A clear decrease of protein density from the centered maximum to the edges of the grid is observed. The fit gives a FWHM of 1 mm and a total particle count of 2.9 × 10^9^. We can compare this number to the estimate from the total accumulated deposition current of 20 pAh. Using the most abundant apoferritin charge state of +50, this corresponds to 9.0 × 10^9^ particles. We attribute the deviation partially to ambiguity of the charge state, due to the continuous mass to charge distribution of ferritin, caused by the randomness of the mass of the iron cores. Hence, the charge state distribution cannot be measured with ensemble MS techniques. This makes the calculation of the number of landed particles less accurate. In addition, apoferritin, which accounts for 40% of the total ion-beam intensity, was not detected due to radiation damage.

The different approaches to the measurements of the deposition spot size provide comparable results and show that the ion beam can be focused to reduce the preparation time of high-density protein samples. Differences in the spot size can be understood by the use of two different proteins, DC potentials, and different sample geometry. The AFM sample is thicker, and thus closer to the front plate. This changes the local electric fields and leads to a different focus. We have observed that the deposition spot size can be tuned most effectively using the DC potential between front plate and sample. The beam can also be defocused to create a more homogeneous distribution across the entire sample. Generally, either full monolayer coverage or few isolated particles can be achieved to optimize the sample for various imaging applications.

The size and shape of the deposition spot measured here is consistent with other observations. Secondary ion mass spectrometry together with infrared reflection absorption spectroscopy showed similar distributions of below- and above-monolayer coverage.^48^ Most importantly, the strong influence of the fields directly at the sample suggest that more effective focusing could be achieved with dedicated ion optics installed at this location.

### Control of Conformation after Landing by Mass Filtering and Solution Composition

It is established, for example by ion mobility spectrometry, that the three-dimensional (3D) conformation of proteins can be retained to a large degree in native ESI.^54^ To study if such a native-like conformation can be retained in our instrument, we soft-landed BSA on HOPG using different solutions and instrument settings.

**Figure 5:**
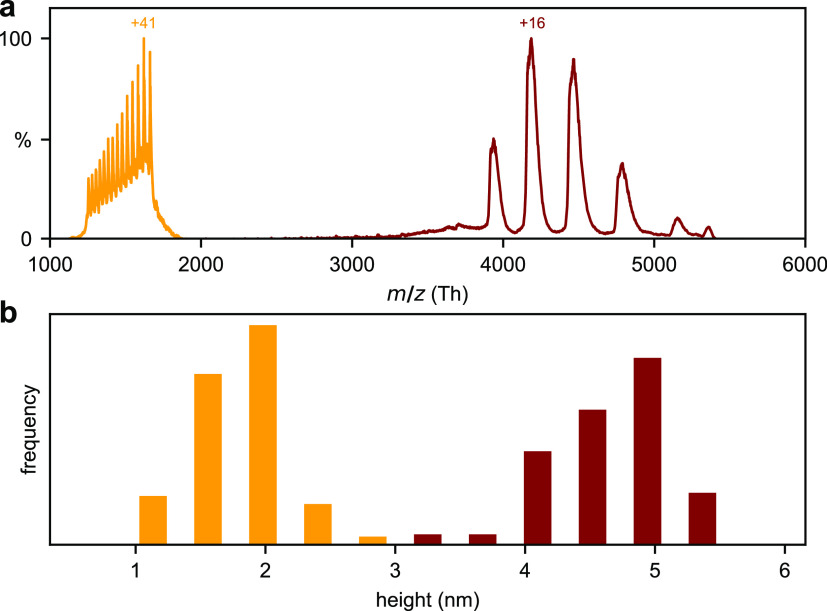
Native BSA (red) and denatured BSA (yellow) mass spectra and height histograms. Spray solution composition: Native 200 mmol NH_4_Ac, denatured 73:24:3 MeOH:H_2_O:HCOOH. (a) Mass spectra for native (filter window 2000–5000 *m*/*z*) and denatured (1250–1700 *m*/*z*) BSA. (b) Resulting height distribution measured with AFM after soft-landing on HOPG. Native BSA sample size = 47, denatured BSA = 60.

Figure 5 shows two mass spectra of BSA. When using a solvent containing 73% MeOH, 3% HCOOH (formic acid), and 24% water and a conventional ESI source, high charge states were observed indicating that the protein is denatured and unfolded. We selected the charge states +40 to +53 with the mass filter for deposition. For a 200 mmol L^–1^ NH_4_Ac solution nanosprayed at 1.2 kV, much lower charge states between +14 and +17 are observed, which indicate folded BSA. We selected only the BSA monomer for deposition.

After deposition, AFM images are taken and quantitatively analyzed (see Methods) to extract the height distribution, which allow an approximation of the shape of the adsorbed proteins. The height distribution is (1.8 ± 0.3) nm for denatured BSA, and (4.7 ± 0.4) nm for native BSA, given as mean ± standard deviation.

Adsorbates originating from highly charged, denatured protein ions appear much flatter than their low-charged native counterparts. This difference in height is consistent with proteins in completely unfolded and globular conformations, respectively. However, it is not possible to directly image the conformation of individual soft-landed proteins in ambient AFM. First, the individual BSA molecules have undergone diffusion limited aggregation^55^ on step edges and terraces. Hence, the individual proteins cannot be identified unambiguously (Figure S5 and Figure S6). Second, the AFM radius of the tip is too large to resolve the lateral shape of the aggregates. Instead, a convolution of the tip shape and adsorbate shape is measured, but the height is reproduced with great accuracy (Å).

This result proves that the ionization conditions, notably source and solvent, control the conformation of the soft-landed protein on HOPG. The CCS describes the ion conformation in the gas phase ahead of the landing event. The CCS of BSA measured in N_2_ for charge state +40 to +53 is 134 to 144 nm^2^,^56^ and for native BSA (+14 to +17) it is 45 nm^2^.^57^ Our measured heights are in good agreement with these values because high CCS, extended denatured conformations yield flatter agglomerates than native, compact ones. Therefore, protein height measurements after soft-landing can reveal prelanding gas-phase conformations on mass spectrometers without IMS capability. This is consistent with previous observations that conformations are retained, on the level of a general shape, after soft-landing on a relatively inert surface like graphite.^18,28,58^

### Mass-Selective Preparation of Cryo-EM Protein Samples

For large, folded protein assemblies, cryo-EM has become one of the leading methods for structural characterization at atomic resolution.^6,7^ Negative-stain EM, on the other hand, is commonly used to screen sample quality before preparation of cryo-EM samples. Native ES-IBD has the potential to complement and accelerate established cryo-EM sample preparation workflows by selective sample preparation and direct correlation between cryo-EM density maps with complementary information about native interactions and small ligands from mass spectrometry.

Our ion-beam deposition instrument can cover TEM grids with mass-selected protein assemblies, with accurate landing energy control, for imaging in negative-stain EM and cryo-EM. Native gas-phase protein ions are generated via native electrospray ionization, then mass selected, and deposited on TEM grids at room temperature. Grids are retrieved via the vacuum load-lock, transferred under ambient conditions, and either stained using uranyl acetate or manually frozen in liquid nitrogen to create cryo-EM compatible samples while circumventing vitrification.

**Figure 6:**
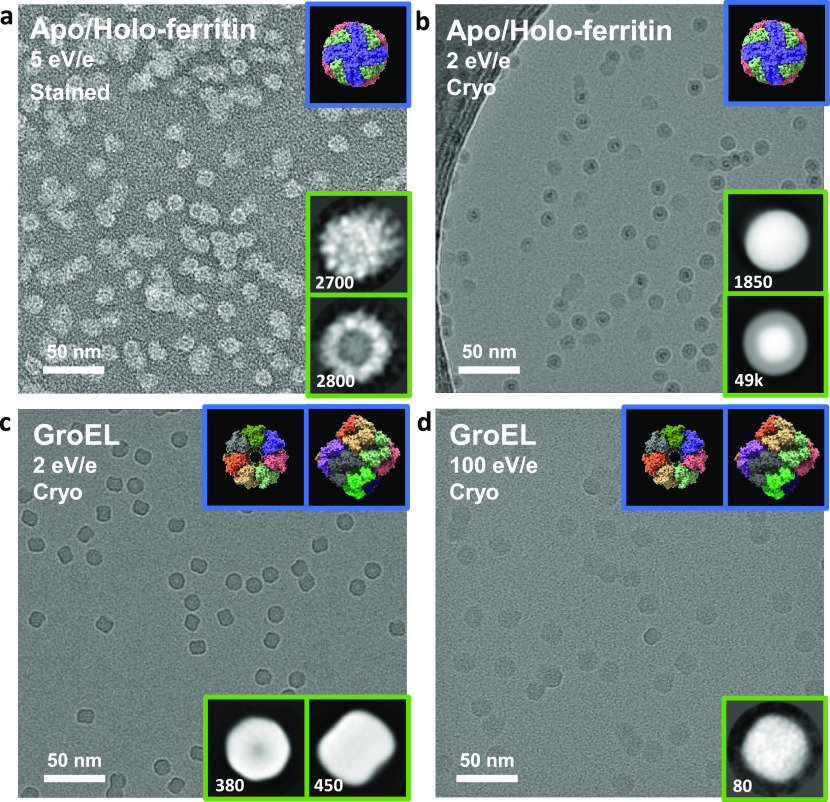
Negative-stain and cryo-EM micrographs of apo/holoferritin and GroEL after gas-phase purification and gentle deposition on TEM grids. (a) Apo/holoferritin, landing energy of 5 eV per charge, 30 nm amorphous carbon film, stained with uranyl acetate. (b) Apo/holoferritin, landing energy of 2 eV per charge, 3 nm amorphous carbon film, plunge-frozen in liquid nitrogen. (c,d) GroEL, landing energy of 2 eV, 100 eV per charge, 3 nm amorphous carbon film, plunge-frozen in liquid nitrogen. The insets show 3D models from the PDB (blue), rendered with ChimeraX^59^ using PDB entries 7A6A for apoferritin and 5W0S for GroEL, and 2D classes of native ES-IBD samples (green) obtained using RELION 3.1. The number of particles in the 2D classes is given in the insets.

Figure 6 shows negative-stain and cryo-EM micrographs from native ES-IBD samples of apo/holoferritin (479 kDa) and GroEL (803 kDa). 3D models from the PDB (blue) and two-dimensional (2D) classes (green) obtained from single particle analysis in RELION 3.1 are shown as inserts.

In the micrograph of a negative-stain sample of an apo/holoferritin mixture, Figure 6a, individual proteins with and without iron cores can be identified. The edges of the protein shell in the 2D classes are less defined than for a control sample made by conventional liquid deposition (shown in Figure S7). The apoferritin 2D class indicates structural heterogeneity, likely due to a deformation of the hollow protein shell, while the holoferritin is stabilized by the presence of the iron core in its center.

Westphall et al. recently developed a similar method to deposit mass-selected proteins with a modified Q-Exactive UHMR mass spectrometer.^30^ They replaced the HCD-cell quadrupole with a sample transfer rod. This setup does not determine the landing energy or control the amount deposited via current measurements.^60^ In contrast to our method, they landed the protein ions in a glycerol matrix before negative staining. Due to this final transfer from gas phase to solution, information about the gas-phase structure is not accessible. Their map of GroEL and other protein complexes shows more detail. This highlights that landing, interaction with the solid substrate, and vacuum exposure can influence the structure of protein complexes, and a high level of control is needed to minimize deviation from native structures.

Combining ES-IBD of protein complexes with negative stain TEM, with or without liquid matrix, has great potential for screening applications. However, we have focused on cryo-EM sample preparation because negative staining ultimately limits access to high-resolution and information on internal structure.

A micrograph of a native ES-IBD cryo-EM sample of the same apo/holoferritin mixture is shown in Figure 6b. The particles have a significantly higher contrast compared to conventional cryo-EM micrographs, due to the use of a 3 nm thin amorphous carbon film and the absence of ice. The ferritin protein shells are clearly visible around the iron cores and demonstrate conservation of protein complex topology. A slight deformation of the apoferritin is still observed, but it is smaller than for the stained sample, and the 2D classes show sharp rather than diffuse edges.

This result indicates that the deformation observed in Figure 6a is not only due to the deposition on dry samples at room temperature, but also due to the influence by negative staining. We suspect that the exposure to the air–water interface in the staining step limits sample quality in this workflow.

Finally we compare ES-IBD samples of GroEL prepared with landing energies of 2 and 100 eV per charge, imaged by cryo-EM, and shown in Figure 6c and Figure 6d, respectively. Top and side projections of GroEL can be identified unambiguously in the sample prepared at the lower landing energy. The features of the characteristic barrel shape, including the central cavity and heptameric symmetry in the top view, are already apparent in the single particle images. Particle dimensions indicate no lateral deviation from literature values. However, further detailed substructure, as observed in samples prepared by plunge-freezing, is not visible. We attribute this to small random changes in secondary and tertiary structure. The changes could be caused by dehydration, landing, and surface interactions. They limit the amount of information that can be obtained by averaging techniques (see Esser et al. for a detailed discussion^29^).

In the sample prepared using a landing energy of 100 eV per charge, Figure 6d, individual particles are still clearly visible, but they are up to 30% larger in diameter, and the distinctive structural features have disappeared. Identification of side and top views is no longer unambiguous. This clearly shows plastic deformation of the GroEL complex due to the energetic impact on the surface, as all other conditions were kept identical. Our workflow enables systematic investigation of the landing energy dependence of this deformation to infer mechanical properties of proteins and protein assemblies.

### Retention of Enzymatic Activity

The difference in structural detail observed between the plunge-frozen cryo-EM samples and ES-IBD samples suggests a level of structural change. To study to what degree this structural change can affect the biological function of proteins, we tested whether the noncovalent protein complex ADH retains enzymatic activity after deposition and resolvation. So far, this has only been shown for recalcitrant single-stranded proteins with no prosthetic groups such as trypsin.^61,62^ We adapted a photometric assay to quantify ADH activity by NADH production after landing on a surface.

**Figure 7:**
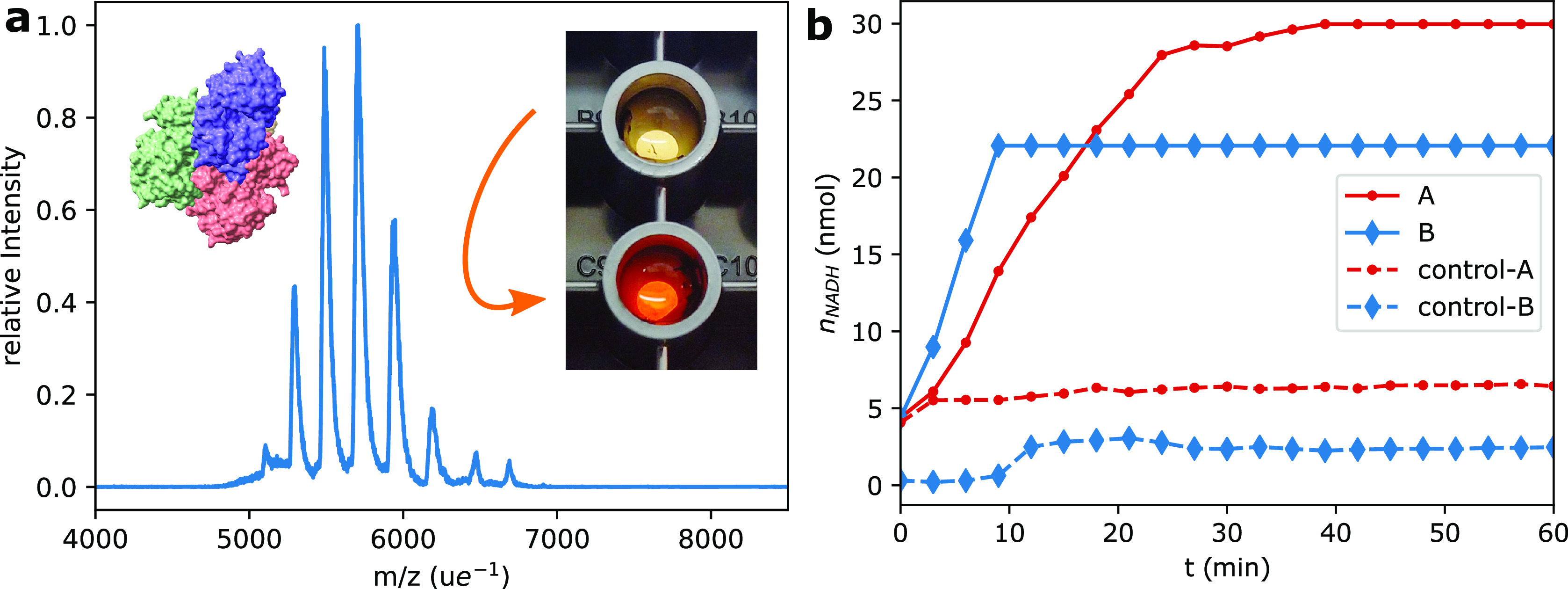
(a) Mass spectra of deposited, mass-selected ADH tetramer. Inset: Color change from yellow to orange indicates an active ADH in the lower well. The black objects on the well’s walls are the submerged ADH-coated conductive tapes. (b) Production of NADH by ADH after ES-IBD. The broken lines stagnating at the offset level are background controls, so the NADH production is specific for ADH activity. The absorbance measurement causes two separate artificial saturation levels due to different calibrations.

We deposited ADH on conductive carbon tapes with 27 ng (128 pAh) ADH for repetition A, and for repetition B with 22 ng (102 pAh). For each experiment two samples were made. Assuming a 2.5 mm diameter deposition spot, this corresponds to two monolayers on average. Figure 7 shows production of NADH by the samples together with background control submerged conductive carbon tapes. The ADH activity is proportional to the slope in of the curves in Figure 7b. It was 1.2 mU (A) and 1.9 mU (B). Minimal (A) or no (B) background activity was recorded in the corresponding time frame. The recovery, based on ADH data sheet activity (300 mU g^–1^), was 14% (A) and 29% (B). When the activity of the spray solution is taken as a reference (A: 88 mU g^–1^, B: 138 mU g^–1^), we find activities of 48% (A) and 65% (B) for soft-landed ADH. The positive control activity was lower than spray solution activity (A: 56 mU g^–1^, B: 117 mU g^–1^). We measured no activity for a 27 ng (128 pAh) conductive carbon tape after 3 days storage in a vacuum (Figure S11). (For further details on attempted ADH extraction, refer to the SI).

These results offer compelling evidence that a large, noncovalent protein complex can survive the entire ES-IBD workflow including ionization, dehydration, transfer into a high vacuum, soft-landing, and resolvation. It is difficult to quantify the exact proportion of intact enzyme. Instead of the numerical value, the order-of-magnitude of the activity is relevant. A number of experimental uncertainties cause this: When reconstituting the commercially obtained, crystalline ADH, it is not known which proportion of the enzyme refolds incorrectly and remains inactive. We measured the spray solution concentration photometrically using a calculated attenuation coefficient. Surprisingly, a much higher proportion of deposited ADH than expected from these references was found to be active. Thus, we used an extrapolation and later a nonlinear calibration (see Methods).

Additionally, the conductive carbon tape could have blocked a small part of the plate reader beam path inside the well and increased absorbance. To mitigate errors, deposited ADH quantity should be cut to a third to remain in the linear range and the reading frequency increased. The loss of all activity after 3 days storage in a vacuum at room temperature might be a consequence of degradation, surface interaction, or desolvation. Further experiments are required to investigate if the soft-landed reconstituted ADH was the intact homotetramer. TEM images of ADH, soft landed under comparable conditions, indicate no fragmentation or change in quaternary structure.^29^

## Conclusion

This work details the conversion of a high-mass range serial Orbitrap mass spectrometer into an instrument for molecular ion beam deposition. A native ES-IBD mass spectrometer requires high beam intensity, ion beam monitoring and control, and adjustable, low, and narrow deposition energy. While existing ES-IBD prototype mass spectrometers show some of the desirable features,^2,9,30,39,52^ such an instrument is currently not commercially available. The focus of this instrument modification is the deposition and imaging of native proteins in order to add chemical selectivity to the protein structure determination process.

Beyond additional ion optics and a deposition stage, it requires the complete understanding of the instruments’ beam handling. This allows aligning of the added components with the duty cycle of the original instrument. To this end, we implemented ion current monitoring at several lenses throughout the instrument. In combination with small modifications to the existing ion optics for better transmission, we obtained a narrow energy-width (≤1.2 eV) native ion beam of sufficient intensity (20 pA). Finally, an intuitive, homemade beam guiding and monitoring software helps to characterize the beam performance and obtaining reliable, reproducible deposition results.

The instrument produced soft-landed protein samples for TEM and ambient AFM analysis. They confirmed the retention of the native-like globular conformation. However, electron density maps from samples prepared with ES-IBD currently lack the necessary resolution to determine the extent of structural change related to the current implementation of the native ES-IBD workflow.^29,30^ As an alternative approach to check the integrity of the deposited protein, we have conducted an enzymatic assay. It indicated that the activity of the noncovalent protein complex ADH was retained post-deposition.

The instrument developed here shows that a commercial platform can be modified for reliable and fully controlled depositions, while the excellent performance of the mass spectrometer is retained. The extended capabilities of the mass spectrometer, such as ion activation or high-resolution selection of a fragment ion, offer interesting perspectives for future experiments.

## Methods

### Mass-Filtered Electrospray-Ion-Beam-Deposition Machine Design

We converted a Thermo Scientific Q Exactive UHMR (Ultra-High Mass Range) into a preparative mass spectrometer (Figure 1). The electrometer at the end of the HCD cell was removed to make space for a custom deposition stage. Analytical tandem MS still works unaffected in the modified UHMR.

The deposition stage contains a 2 × 8 element electrostatic lens to focus the ion beam. Steering lenses deflect the beam laterally to any position on the sample holder. A 2 mm diameter aperture separates the two lens stacks. The first lens stack is pumped via the Q Exactive UHMR quadrupole. A 67 L s^–1^ turbo pump in the deposition part pumps the second part (HiPace 80, Pfeiffer Vacuum GmbH, Asslar, EU). A CF 40 gate valve (series 01, VAT Vakuumventile AG, Haag, Switzerland) decouples the deposition stage from the analytical mass spectrometer. After the gate valve, an immersion lens shields the ion path from the electric potential of the grounded vacuum chamber. Hence, beams with negative total energy (*E*_tot_) vs GND can pass.

The sample holder has two sample positions for EM grids or AFM samples and an energy detector to measure beam *E*_tot_. A custom sample transfer stick moves it from a load lock to high vacuum (HV). RBD 9103 HV floating picoampmeters (RBD Instruments Inc., Bend, USA) measure ion current on aperture, sample holder front plate, samples, and the energy detector. An ECH 244 crate with 2× EBS 180 ± 500 V bipolar power supply insets control all DC voltages to deposition stage (ISEG Spezialelektronik GmbH, Radeberg, EU). Home-written control software for the picoampmeters and power supplies facilitates the ES-IBD workflow. It supports rapid 2D ion beam imaging, *E*_tot_ beam measurement, and automatic beam focusing optimization.

To use sweep gas with the nano-ESI source, we milled a 20 mm bore in the cone gas adaptor. The S-lens diameter was increased from 1.4 mm to 2.0 mm and later to 2.5 mm to improve ion transmission. Consequently, gas throughput at the source turbo pump (Splitflow 310, Pfeiffer Vakuum GmbH, Asslar, EU) rose from approximately 2.7 mbar L s^–1^ to 5 mbar L s^–1^. We separated the fore pump system to protect the Splitflow 310. The S-lens chamber remained pumped by the factory-fitted Sogevac SV65BiFc fore pump (Atlas Copco, Stockholm, EU), and the Splitflow 310 was connected to an Edwards XDS 35i (Atlas Copco, Stockholm, EU) fore pump. This increases the pressure in the inter-flatapole chamber but does not affect the pressure in the C-Trap or Orbitrap mass analyzer.

### Deposition Workflow

The first step is to load two EM-grid or AFM highly oriented pyrolytic graphite (HOPG) targets into the sample holder. The transfer rod moves them from the ambient load lock to the high vacuum deposition chamber. While the pressure therein decreases, we prepare the ion beam.

For native proteins, we use gold-coated 1.2 mm glass capillary emitters. We select the minimum possible pressure to push the spray solution to the tip. This maximizes emitter lifetime. We start the instrument in normal analytical configuration to check if the emitter is working. We set the mass filter window, then switch to beam mode. Both samples are kept at a high, repulsive potential to avoid uncontrolled deposition. In beam mode, the C-trap and the HCD cell guide the ions without pulsing into the landing stage. All DC potentials within the Q Exactive UHMR instrument are at the default values to guarantee activation-free transmission from source to the deposition stage. In contrast, analytical native MS typically uses strong gradients, often in pulsed modes, to desolvate or dissociate protein complexes.^63^ HCD gas flow is set to 7 to thermalize the ion beam in there.

To optimize the current, we change the emitter distance, backing gas pressure, and the cone gas flow. If the current is sufficient for deposition, we switch to analytical mode and acquire mass spectra of the ion beam. The instrument is set beam mode again and the beam steered on the energy detector. The detector has a metal grid in front of the collector plate used to measure current. If the electric potential on the metal grid is higher than the total beam energy, the ions cannot pass. Hence, we record the detector collector plate current as a function of the grid potential to obtain the beam energy.

Then, we select the retarding potential on the sample. The difference between the beam energy and the retarding sample potential determines the landing energy, typically 5 eV per charge. We deflect the beam on the sample and start the sample current integration. Once the charge reaches the defined value, the repulsive potential is reapplied. The beam composition is periodically controlled using the mass analyzer, including every time we replace the nanospray emitter. The TEM imaging deposition procedure has been already described.^29^

### Energy Width

We used a native and a denatured BSA beam. For preparations see below. All DC voltages within the Q Exactive UHMR instrument were at the default values. For both beams, we applied a weak or strong DC gradient in the landing stage optics. This focused them through the electrostatic lens on the energy detector. Figure S1b shows the different voltages applied to produce a weak or strong gradient.

The voltage on the detector metal grid was swept in 40 voltage steps around the expected beam-energy value. For every voltage step, we recorded the average of 60 detector current measurements. This dampens arbitrary or short-term periodic current fluctuations. The negative differential of the current by the voltage was fitted with a Gaussian distribution. The fit gives the mean beam energy and its fwhm.

### Transmission

To measure ion current within the Q Exactive UHMR instrument, we added breakout cables. To this end, we separated the transfer capillary voltage supply from S-exit lens. Breakout cables were connected to the S-exit lens, the inter-flatapole lens, the inner Turner–Kruger (TK) lens, and the HCD exit lens. A modified cone gas cap adaptor supplies the transfer capillary voltage. Each breakout cable connects a RBD 9103 picoampmeter to a DC ion optic and the corresponding power supply on the Q Exactive UHMR DC supply board.

For the current measurement in the Q Exactive UHMR, we set the DC optic (e.g., the S-exit lens) to an attractive potential and the following RF ion optics axis DC (e.g., the injection flatapole) to a repulsive potential. This ensures the entire beam is collected on the DC optic in question. All voltages are in Table S1 in the SI. In the deposition stage, we deflected the beam instead on the aperture or energy detector.

In the next step, we moved the emitter sidewards away from the transfer capillary to block the ion beam at a preceding element. The current offset was recorded and the emitter moved back in position. Then, we recorded the current. All values in Figure 3 are offset corrected.

We used the heated ESI source for Rhodamine B and denatured BSA solutions. The nano-ESI source was used for native Ferritin and native BSA.

### Ion Beam Shape Analysis and Control

#### 1 On a Front Plate

We obtained a 2D image of the front plate with a denatured BSA beam. We chose denatured BSA, as it reproducibly provides an intense and stable ion beam, which allows collection of high-quality images. To obtain a scanned image, we deflected the beam horizontally and vertically with the steering lenses while recording the current on the front plate. See Figure S14 for an explanatory scheme. A 41 × 65 pixel scan was obtained in 34 min. The image dimensions were then converted from volts to millimeters by calibration with the actual front plate size. The image represents a convolution of the sharp front plate geometry, a function of only 0 and 1, and the ion beam profile, assumed to have a Gaussian shape. We used a Python script to deconvolute. It employs a binary filter to create a sharp version of the image and then applies the convolution theorem to obtain the beam profile. Finally, we used a low pass filter to remove high frequency components, originating from the nonperiodic image boundary.

#### 2 On a HOPG AFM Sample

We deposited 12.5 pAh of GroEL and used a NanoScope MultiMode AFM for imaging. GroEL was prepared as described in subsection Spray Solution Preparation. For deposition, we followed the standard workflow. Except for the front plate voltage. It was at −10 V, as close as possible to the beam energy of −7.0(16) eV per charge, to minimize the deposition spot size. We acquired multiple 5 × 5 μm^2^ images on a raster around the deposition spot to further assess the protein distribution. We used the dimensions of the cantilever to raster across the surface and reconstruct a density map. We manually counted the number of aggregates in each image.

#### 3 On a TEM Sample

20 pAh ferritin were deposited on an amorphous carbon TEM grid (AGS160-4, Agar Scientific, Stansted, Great Britain). The front plate was at −20 V to the standard work-flow focus. We used a mixture of apoferritin and holoferritin to obtain high contrast. Under the given conditions only holoferritin iron cores are visible. TEM images were recorded using an FEI Talos 200c at room temperature, and under the given conditions only the holoferritin iron cores are visible. A python script was used to count the number of particles on the TEM images. Measuring the current on the sample for mass-selected apoferritin and ferritin, the ratio between them was determined as 40:60, and the particle counts were corrected accordingly. The density was determined on multiple grid squares as the average of particle counts of three images, divided by the image area. The coordinates of the individual grid squares were obtained according to the grid square size on a 400 mesh TEM grid.

### Spray Solution Preparation

We purchased rhodamine B (R6626-25G), bovine serum albumin (BSA, A0281-1G), equine spleen ferritin (F4503-25MG), GroEL (chaperonin 60, C7688-1MG), and baker’s yeast alcohol dehydrogenase (A7011-15KU) from Sigma-Aldrich (Darmstadt, EU). Ferritin and GroEL preparation has been already described.^29^ We dissolved rhodamine B in 80:20 H_2_O:iPr to 1 × 10^–4^ mol L^–1^. We made a denatured 4 × 10^–6^ mol L^–1^ BSA solution for AFM deposition in 73:23:3 MeOH:H_2_O:HCOOH. For all other denatured BSA measurements, we used a 3 × 10^–6^ mol L^–1^ 100:100:1 ACN:H_2_O:HCOOH solution. We desalted native BSA and ADH twice with size-exclusion chromatography columns (P6, 7326222, Biorad, Hercules, USA). These were equilibrated with 0.2 mol L^–1^ ammonium acetate (A2706-100 ML, Sigma-Aldrich). Resulting concentrations were 2 to 5 × 10^–6^ mol L^–1^. For all preparations, deionized water with ρ ≥ 18.2 MΩ m filtered through 0.22 μm was used. All other solvents were MS grade from changing suppliers.

### AFM Analysis

Prior to deposition, each highly oriented pyrolytic graphite chip (HOPG, MikroMasch, Sofia, EU) was cut into 5 × 5 mm chunks and glued with leit-silver (09937, Sigma-Aldrich) on an AFM stainless steel support. We used a multimode AFM (AS-Micro, Indianapolis, USA) with a Scout 350 silicon tip (Nunano, Bristol, Great Britain) in tapping mode at room temperature. The AFM images were further processed with Gwyddion. We used the graphite step-edges for height calibration. We selected the highest point of each protrusion as height measurement.

### TEM

Ferritin (F4503-25MG) and GroEL (chaperonin 60, C7688-1MG) samples were purchased from Sigma-Aldrich. Sample preparation was carried out using a standard native MS workflow, including exchange of buffer to volatile ammonium acetate, as described before.^29^ All samples were imaged using a Talos Arctica 200 kV (Thermo Fisher Scientific), and images were processed using RELION 3.1, as described in Esser et al.^29^ For staining, 30 nm amorphous carbon TEM grids (AGS160-4H, Agar Scientific) were plasma cleaned before deposition. After deposition, dry grids were placed on 25 μL of 2% uranyl acetate, blotted, and left to dry. A control sample was prepared by applying 4 μL of 10 μM ferritin in PBS to the grid for 2 min, followed by blotting, washing, and staining as described above.

### Retention of Enzymatic Activity

The workflow we developed combines ES-IBD with an adapted photometric alcohol dehydrogenase detection kit (ab102533, Abcam, Cambridge, Great Britain).

#### Principle

ADH-catalyzed oxidation of propan-2-ol yields NADH and propanone: NAD^+^ + propan-2-ol  NADH + propanone. NADH reacts with a colorimetric probe to form a bright yellow complex analyzed at λ = 450 nm. While the manufacturer does not specify the exact mechanism of the kit, it is most likely based on the WST-8 to WST-8 formazan reaction.^64^

#### Preparation

All microcentrifuge tubes and pipet tips were normal PP. All solutions were shielded from direct light and kept on ice, except where mentioned. Each kit was reconstituted according to the manual,^65^ divided into 4 aliquots, and refrozen at −20 °C. On the day of the deposition, we thawed one kit aliquot and the desalted ADH spray solution. We prepared two positive control ADH solutions from crystalline ADH in the supplied buffer to theoretical in-well activities of 4 × 10^–10^ mol min^–1^ and 4 × 10^–9^ mol min^–1^. Reaction mix and background control solutions were prepared as in the manual and kept at room temperature. All solutions were prepared for a 150 μL total volume in well. This is made up of 50 μL active solution (buffer for blank, buffer for extraction, or positive control) and 100 μL of either reaction mix (with substrate propan-2-ol) or background control mix (no substrate). We measured the ADH spray solution absorbance and determined the concentration with a calculated absorbance coefficient of 195 440 L mol^–1^ cm^–1^. Based on this concentration we prepared spray solution positive controls with the same theoretical in-well activity as the other two positive controls.

#### Deposition

We cut a conductive carbon double-sided tape (EM-Tec CT6, 15-000406, Labtech, Heathfield, Great Britain) in half. We removed two-thirds of the protective film on the back and glued it to a stainless-steel AFM support. The entire protective film on the top side was removed and the target installed in the sample holder. We prepared two targets per repetition, one for the sample and one for the background control. To minimize contamination, we immediately installed the sample holder in the deposition vacuum chamber. The deposition followed the standard procedure. We filtered the beam to the ADH tetramer (5000–7000 *m*/*z*, see Figure S13 in SI). The nanospray needle was protected from direct light. We deposited two tapes with 27 ng (128 pAh) ADH for both repetition (A) and (C). For repetition (B), we deposited two tapes with 22 ng (102 pAh). The mass was determined based on the total deposited charge, most abundant charge state, and molecular weight of ADH. In repetition (B), the deposited amount was lower due to low sample current. The landing energy was 5 eV per charge.

#### Submersion and Measurement

The entire kit except for the reaction mix/background control solutions was reverse-pipetted in a 96 Corning 3881 nonbinding surface half area well plate (Corning Inc., Corning, USA). We were doing this in parallel to deposition of the second ADH target. This minimizes both the time the deposited ADH targets spend in a high vacuum and the time they are exposed to the atmosphere. Repetition (C) targets were left for 3 days in the high vacuum deposition chamber. Then, we put the two deposited tapes in a well with their empty side facing the wall and the center optical path free. The wells were already filled with 50 μL assay buffer to avoid gluing the tapes to the well’s wall. We added reaction mix or background control mix and closed the plate with a transparent lid. A FLUOstar Omega plate reader (BMG LABTECH GmbH, Ortenberg, EU) incubated the sample at 37 °C and read absorbance at 450 nm every 3 min for 2 h.

#### Data Analysis

The initial slope of the NADH production (repetition (A): minute 3–15, (B): minute 0–6) was used for activity calculation. We subtracted background activity only if it was positive. Due to the high proportion of active ADH after deposition, we had to extrapolate the linear calibration for repetition (A) in the absorbance range from 1.6 to 2.5 (18 nmol NADH). To attenuate arising errors, we extended a nonlinear calibration for repetition (B) to 2.7 (15 nmol NADH).

## Supporting Information Available

Detailed explanation of the factors influencing ion beam energy distribution, the voltages within the instrument and landing stage, exemplary AFM and TEM graphs used for the deposition spot size calculation, a scheme of the 2D-scan used to characterize the ion beam diameter, AFM graphs of denatured and native BSA on HOPG, a negative stain control TEM image of apo/holoferritin, and additional information about the ADH activity experiment (PDF)
