# Supplementary material for: A Preparative Mass Spectrometer to Deposit Intact Large Native Protein Complexes
Source: ACS Nano. 2022 Aug 29;16(9):14443–55. doi: 10.1021/acsnano.2c04831 (PMC9527803; doi:10.1021/acsnano.2c04831)
Supplement: Supplementary file 1 — nn2c04831_si_001.pdf [file nn2c04831_si_001.pdf]

Supporting information: A preparative mass spectrometer to deposit intact large native protein complexes

Paul Fremdling,[†] Tim K. Esser,[†] Bodhisattwa Saha,[†] Alexander A. Makarov,[‡,¶] Kyle L. Fort,[‡] Maria Reinhardt-Szyba,[‡] Joseph Gault,[†,∥] and Stephan Rauschenbach*,[†,§]

†*Chemistry Research Laboratory, Department of Chemistry, University of Oxford, 12 Mansfield Road, Oxford OX1 3TA, United Kingdom*

‡*Thermo Fisher Scientific, Bremen, 28199, Germany*

¶*Biomolecular Mass Spectrometry and Proteomics, Bijvoet Center for Biomolecular Research and Utrecht Institute for Pharmaceutical Sciences, University of Utrecht, Padualaan 8, 3584 CH Utrecht, The Netherlands*

§*Max Planck Institute for Solid State Research, Heisenbergstrasse 1, Stuttgart DE-70569, Germany*

∥*Present address: Vertex Pharmaceuticals, 86-88 Jubilee Avenue, Milton Park, Abingdon, OX14 4RW, United Kingdom*

E-mail: stephan.rauschenbach@chem.ox.ac.uk

Beam energy

The ion beam is thermalised in the HCD cell at approx. 10^{-2} mbar. The total ion beam energy (TE) is close to the effective potential therein ($-5\,\mathrm{eVz^{-}1}$). When the ions leave the

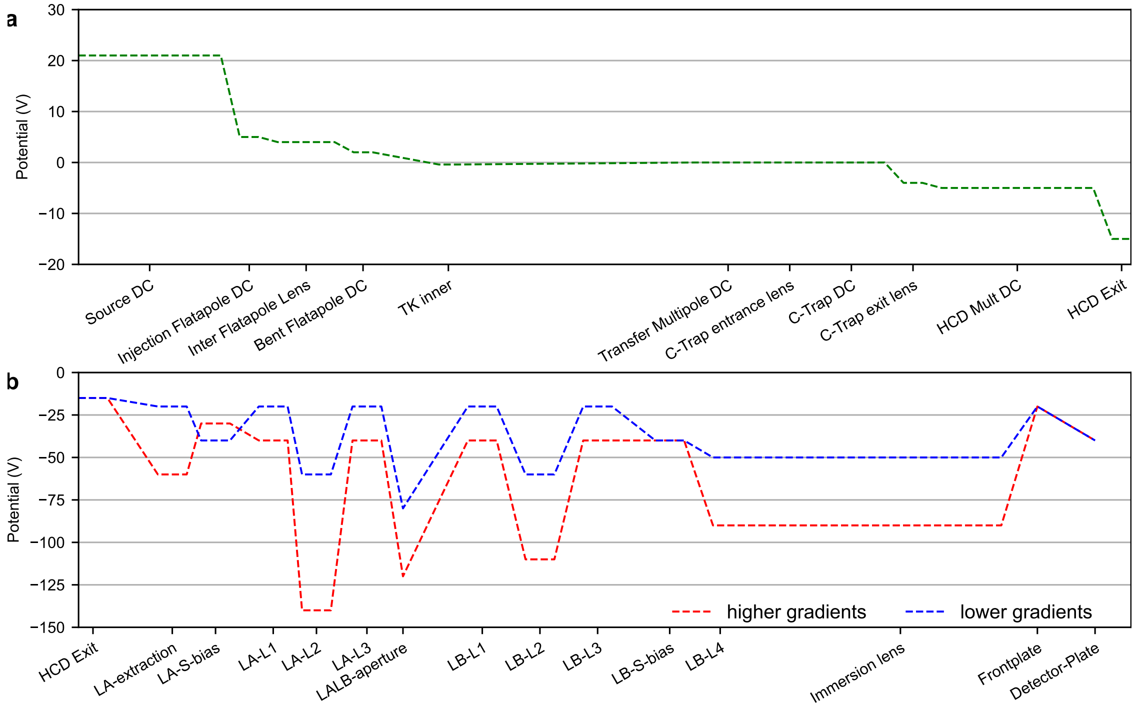

Figure S1: DC Potentials applied within **a** the mass spectrometer and **b** the custom landing stage for higher and lower DC gradients.

HCD cell, they are accelerated in the electrostatic lens. In there, the pressure decreases from high 10^{-3} mbar (HCD side) to 10^{-6} mbar (deposition chamber side). As the electrostatic lens is longer (60 mm for high-pressure part) than BSA mean free path (0.1 mm for a native BSA^{+14} ion at 7×10^{-3} mbar), collisions with the background gas occur. For a hard-sphere collision, the kinetic energy E' of an ion after the collision is:[1–3]

$$\frac{E'}{E} = \frac{m_1^2 + m_2^2}{M^2} + \frac{2m_1m_2}{M^2} \cdot cos(\theta_{cm}) \tag{1}$$

where θ_{cm} is the scattering angle in centre-of-mass coordinates, m_1 the ion mass, m_2 the gas molecule mass, $M = m_1 + m_2$ and E the pre-collision ion kinetic energy. As $m_1 >> m_2$, the second term is close to zero. Thus, equation 1 predicts E' is a fraction of E depending mostly on the ion and gas mass. Fig. S2 compares this effect for heavy and light ions. The decrease in ion kinetic energy $E - E'$ is bigger for higher E. E in the lab frame is proportional to the potential within the electrostatic lens. E maximum is 135 eVz^{-}1 (strong gradient). As

only 8.8 eVz^{-}1 (denatured) respectively 5.2 eVz^{-}1 (native) are dissipated in the electrostatic lens (Fig. 2 "Beam-energy distribution" in main article) , few high energy collisions occur. For each E' is close to the initial kinetic energy. Therefore, the E_{tot} is lower after passing through stronger gradient conditions.

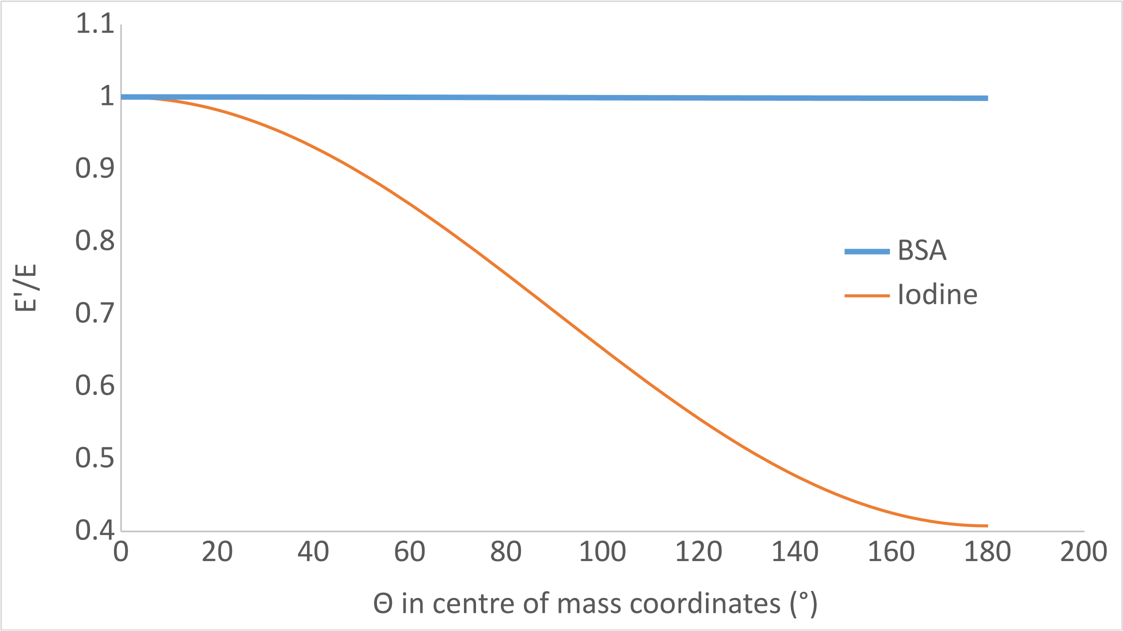

Figure S2: Kinetic energy loss per collision in N_2 for BSA (mass 66 500 u) and Iodine (mass 127 u) as a function of the scattering angle. Minimum for BSA is 0.99832 at 180°.

A similar argument applies to the distribution width: Arbitrary variations of the impact angle between a gas molecule and an ion cause variations in the scattering angle. Term 2 in equation 1 varies accordingly and again E' is a fraction of E, meaning absolute variations in ion kinetic energy $E - E'$ are bigger for higher E.

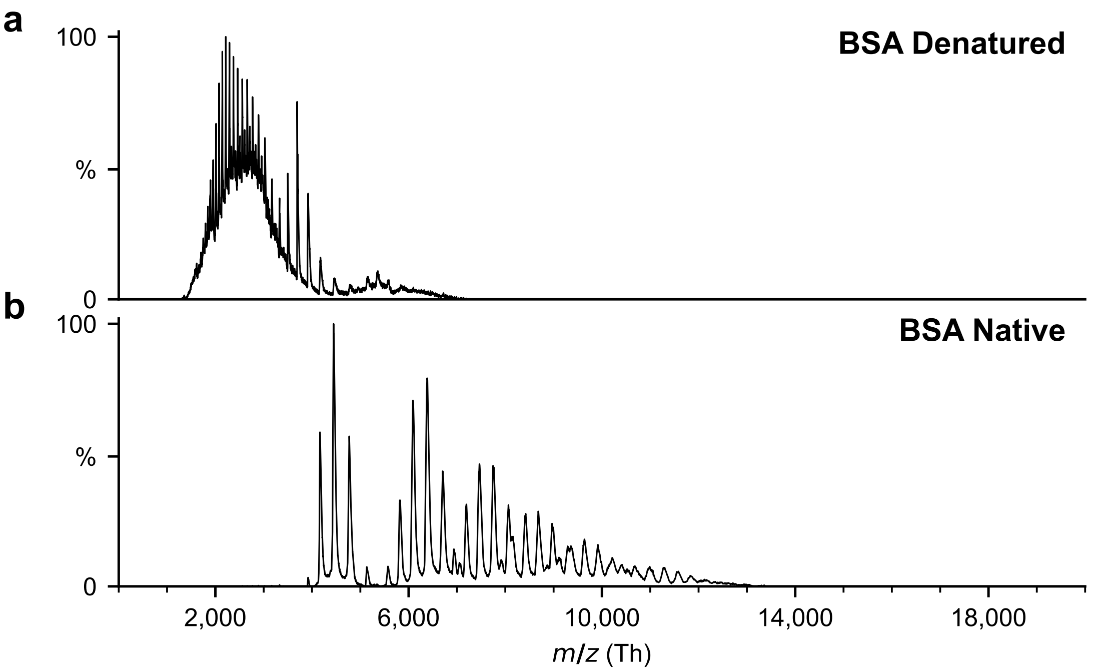

Figure S3: Mass spectra of **a** denatured BSA and **b** native BSA. Both mass spectra are acquired with non-activating conditions used for deposition.

Transmission

Native BSA emission current for the +15 charge state and a nano-ESI flow rate of 1 µlh − 1

$$I = \frac{cVzF}{t} = \frac{3 \times 10^{-6}\,\mathrm{mol\,L^{-1}} 1 \times 10^{-6}\,\mathrm{l} \cdot 15 \cdot 96\,485\,\mathrm{C/mol}}{3600\,\mathrm{s}} = 1.2\,\mathrm{nA} \quad (2)$$

With concentration c, Volume V, number of charges z, Faraday constant F and time t.

Table 1: Potentials applied to ion optics when measuring current. Emitter potential is for nano-spray setup.

**Measure at**	**Φ Optic (V)**	**Repulsive Optic (RO)**	**Φ RO (V)**
Emitter	≈1200	n.A.	n.A.
Transfer capillary	21	n.A.	n.A.
S exit lens	-100	Injection flatapole DC	50
Inter flatapole lens	-50	Bent flatapole	50
Inner TK lens	0	Outer TK	60
Aperture	-60	steer beam	n.A.
Current detector	-20	steer beam	n.A.

Ion beam size

SIMION simulations are consistent with the observations in figure 4 "Ion Beam Shape analysis" in main article. However, since the angular velocity distribution of the beam upstream of the sample holder cannot be determined experimentally, a quantitative comparison of simulated and observed beam profiles is currently not meaningful.

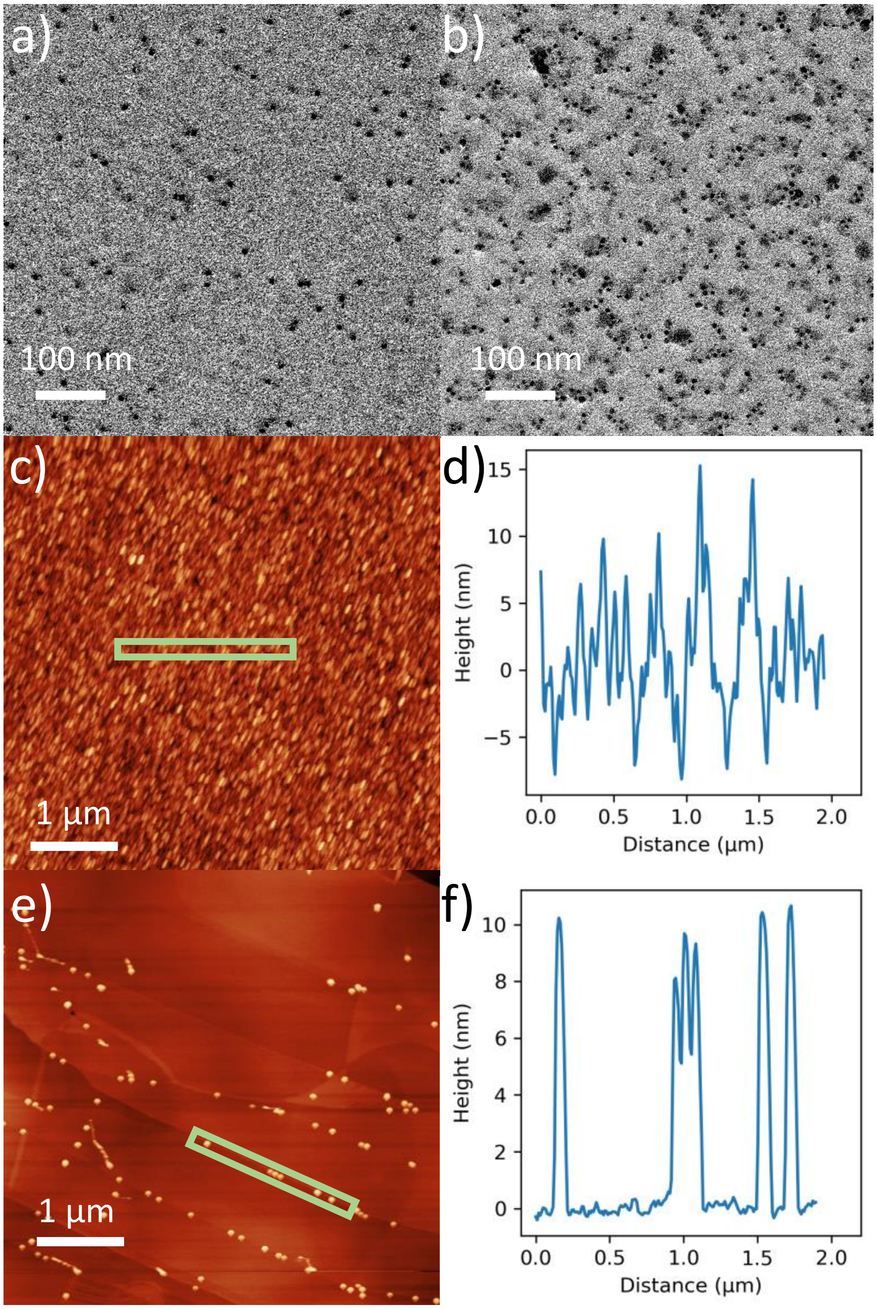

Figure S4: Exemplary AFM and TEM data for the beam shape characterization. **a)** and **b)** show low (250 particles per μm^2) and high (1500 particles per μm^2) density areas on the TEM grid. **c)** shows an area in the centre of the deposition spot with more than monolayer coverage. Therefore, the line profile **d)** shows no well-defined baseline. **e)** shows a less dense area with 4 particles per μm^2. The corresponding line profile **f)** shows a clear baseline and allows to determine the particle heights.

Mass filtering and solution composition

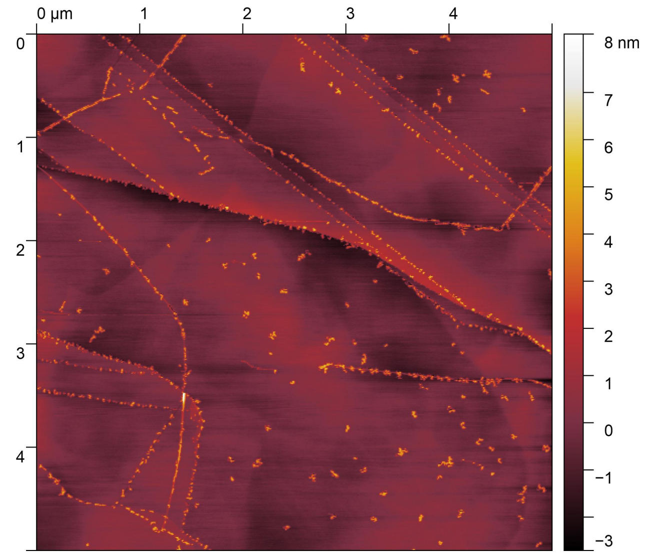

Figure S5: Native BSA on HOPG imaged with ambient AFM

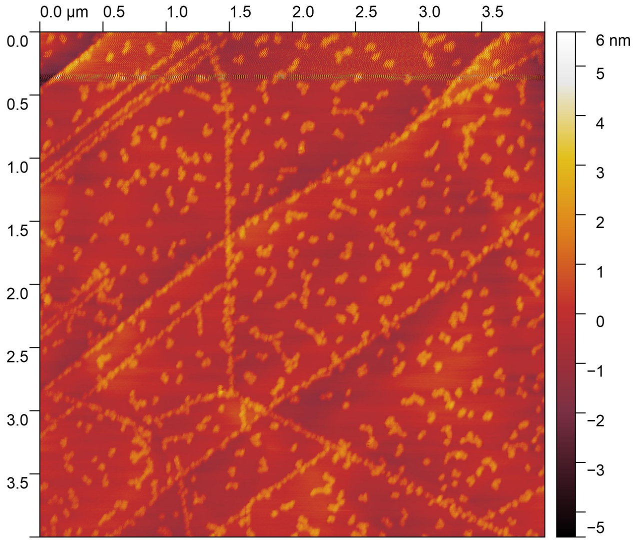

Figure S6: Denatured BSA on HOPG imaged with ambient AFM

TEM

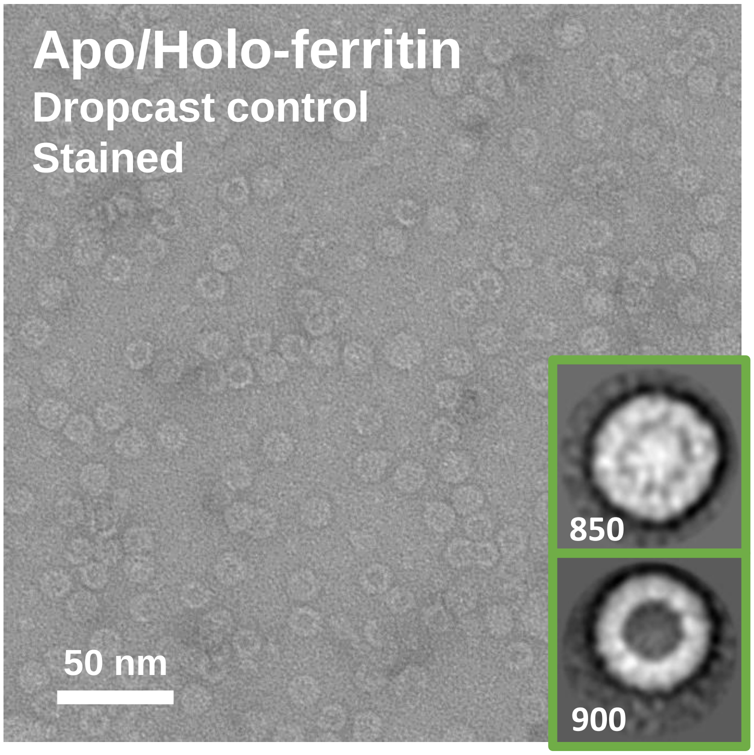

Figure S7: Negative stain apo/holo-ferritin control sample. The 2D classes show the same characteristic features as observed for the native ES-IBD samples, though they are better defined and there is less deformation in the control, despite the lower number of particles.

ADH activity

Method for extraction: Before we established the successful protocol, we tried to extract the deposited ADH. All steps were the same as described for the submersion, except: We deposited two times 27 ng (128 pAh) and two times 37 ng (175 pAh) on four amorphous Carbon EM Grids (AGS160-4H, Agar Scientific Ltd, Stansted, Great Britain). Each 27 ng grid was left for 2 days after deposition in ambient conditions. Then, we put in 50 µl assay buffer in a PP micro centrifuge vial. One 27 ng grid was vortexed for 15 min, the other sonicated for the same time. Then, the we transferred the extract in the 96 well plate. For the 37 ng grids, both were transferred immediately after deposition in a well with 50 µl assay buffer. We moved them around for 6 min with tweezers to wash ADH off. Then, we removed the grids.

Results: Neither sonication- nor vortex- nor washed-off-extracted ADH from EM-grids was active (Fig. S8). Submersion of the EM grid was not possible due to high background activity. We attribute this to a redox-reaction between the kit's components and the grid's copper support, which turned dull. Submersed conductive carbon tape was inert and used for all further work (Fig. S9). To check if the adapted assay protocol was working, we used the nano-electrospray source to deposit two tapes at atmospheric pressure. Sample activity was 2.1 mU, no background activity was present (Fig. S10). Although charge can be measured, recovery calculation is not possible due to unknown composition of the ion-droplet-plume.

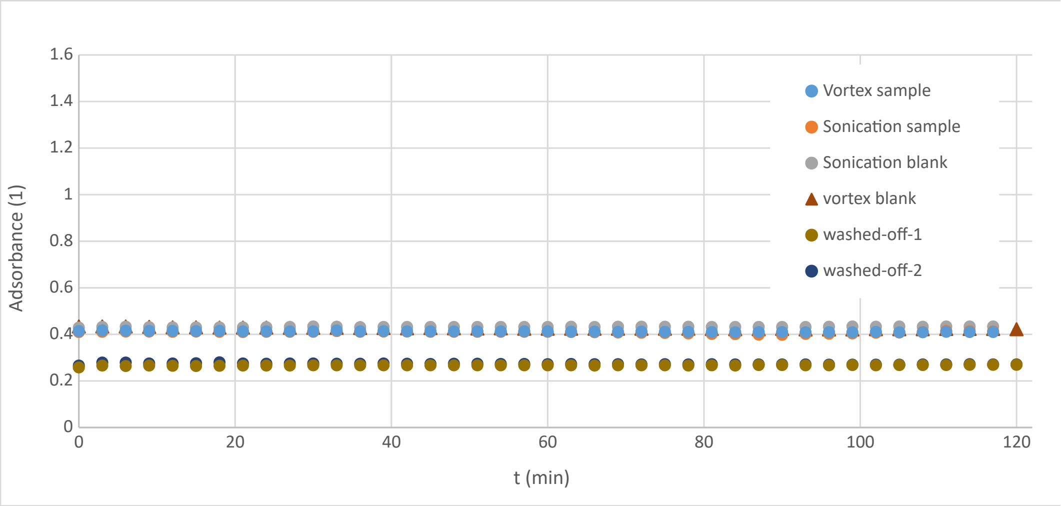

Figure S8: Assay-buffer-extraction of 128 pAh deposited ADH on EM grid with either vortex or sonication yields blank activity. The same applies for washing-off 175 pAh deposited ADH on EM grids. We tried to wash ADH off by moving the deposited grid around with tweezers in ADH buffer.

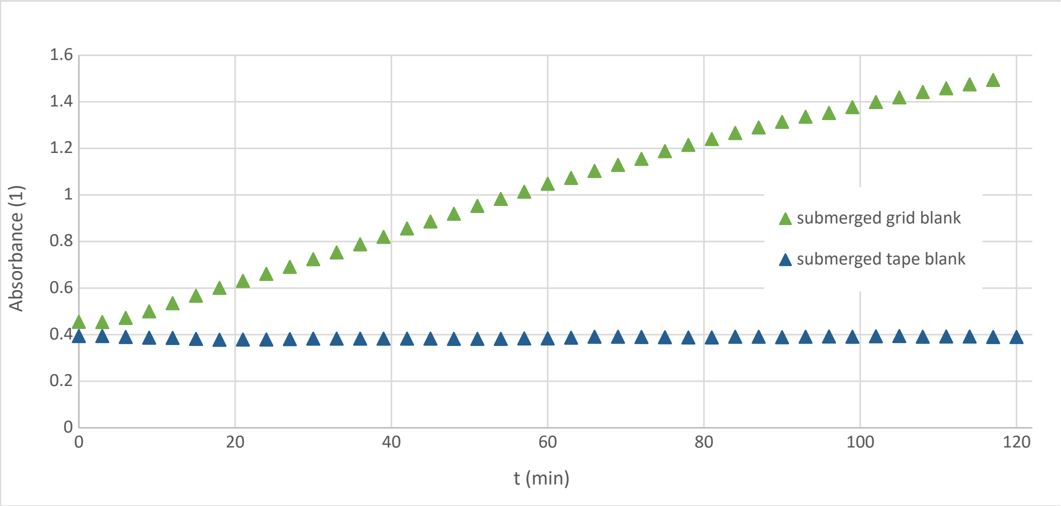

Figure S9: Submersion of an EM-grid in assay reaction mix causes a strong increase in absorbance, conductive tape doesn't.

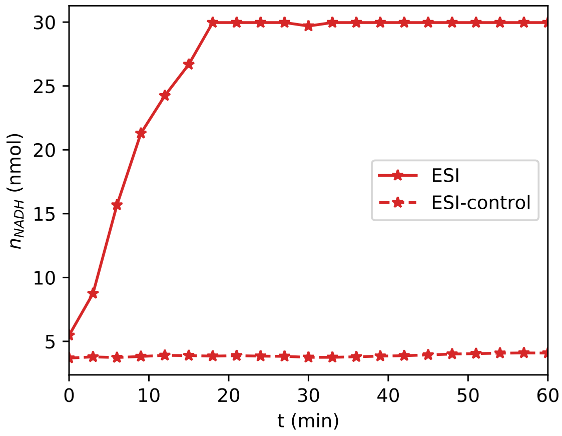

Figure S10: Production of NADH by ADH after electrospray deposition at atmospheric pressure. The broken lines stagnating at the offset level are background controls, so the NADH production is specific for ADH activity.

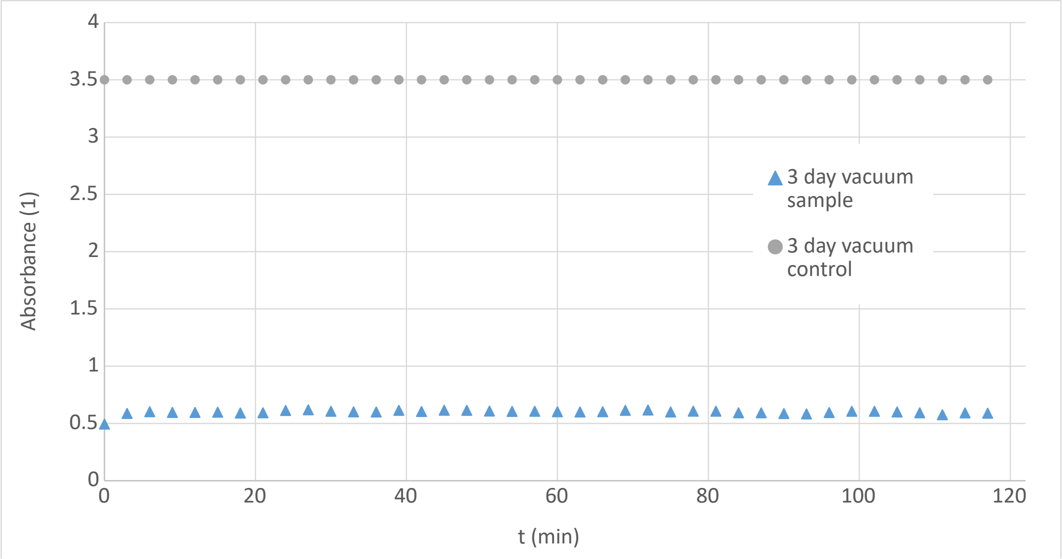

Figure S11: 128 pAh deposition (repetition C) retains no activity after 3 day storage in vacuum. The corresponding control was inactive as well, but showed a high absorbance due to the conductive tape having moved into the beam path (see Fig. S12).

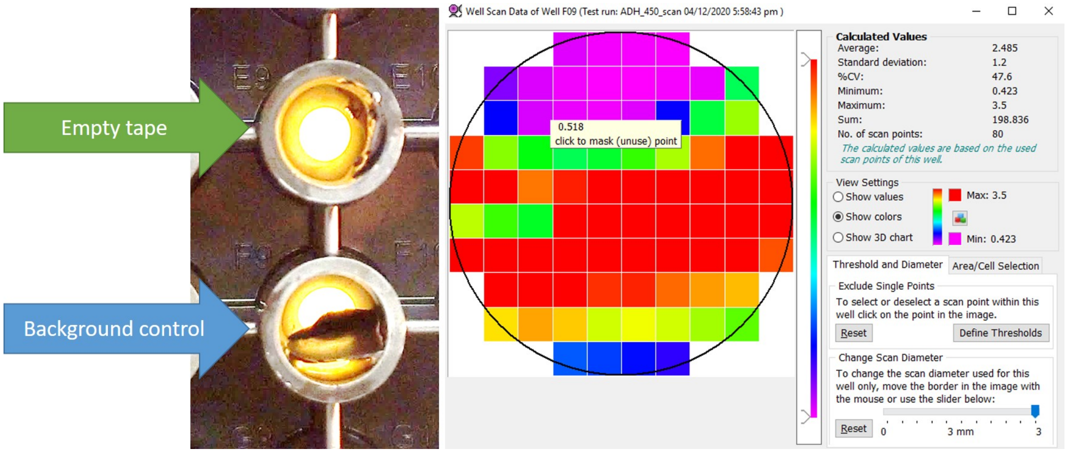

Figure S12: Left: 3 day storage background control (repetition C) moved into plate reader optical path. Note the liquid is still yellow, meaning no reaction has occurred during incubation. Right: 2D scan of bottom well confirms absorbance is still at blank level after incubation.

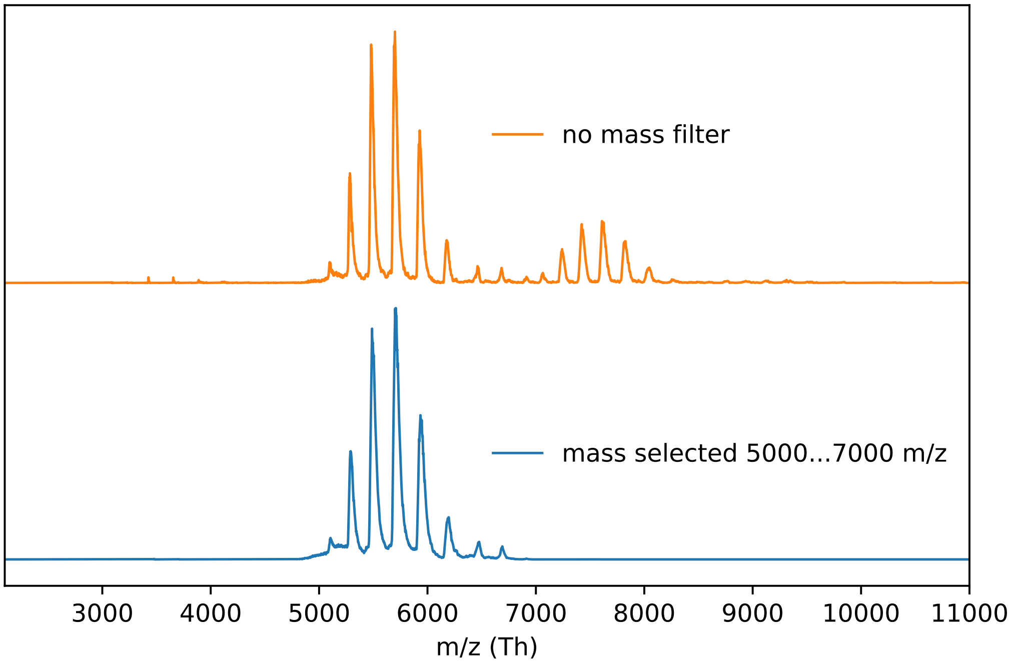

Figure S13: Mass spectra of the ADH beam prior and after 5000...7000 m/z filtering. The mass-selected beam was used for deposition.

2D scan scheme

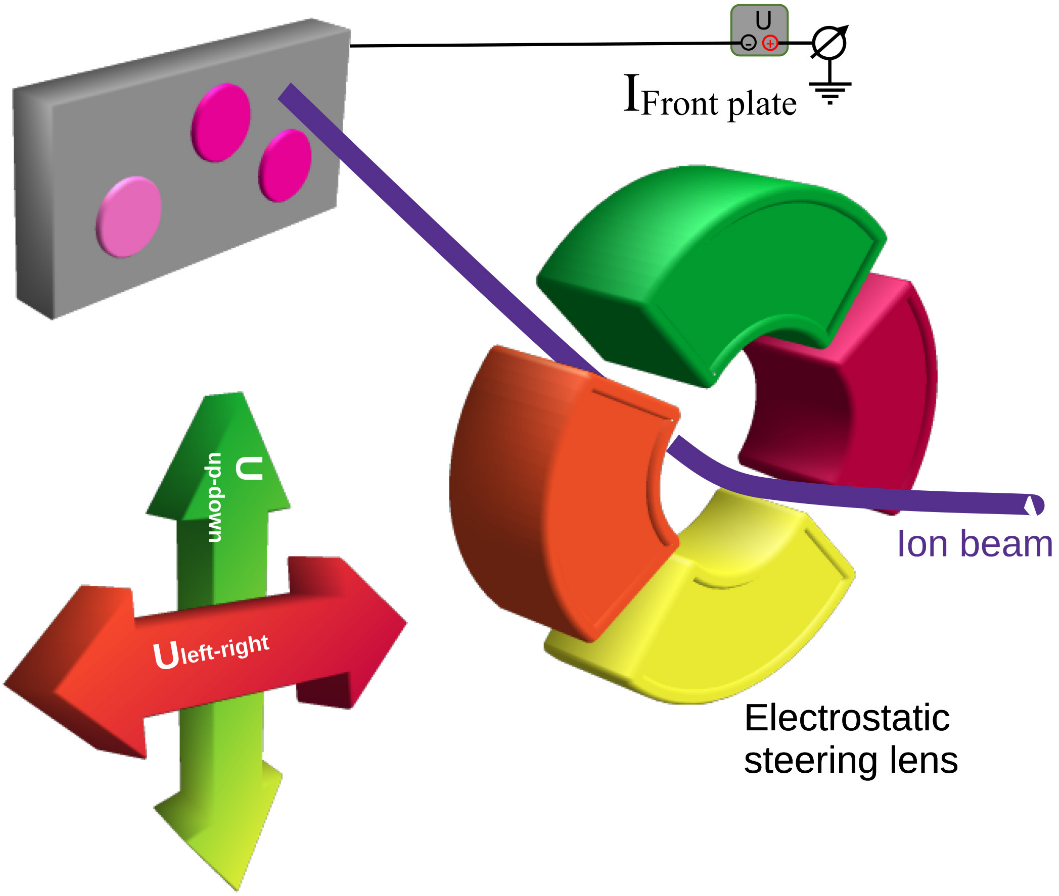

Figure S14: Scheme of the 2D-scan used to characterise the ion beam diameter on the front plate. A single picoammeter measures the current on the sample holder front plate. The data is recorded as a function of the deflection voltages applied to the electrostatic steering lens. The recorded current data can be represented as a 2d map. This shows an ion current image of the front plate. The 2D-scan function is also routinely used to find the sample (dark pink) and energy detector (light pink) positions.

References

1. Douglas, D. J.; French, J. B. Collisional Focusing Effects in Radio Frequency Quadrupoles. *J Am Soc Mass Spectrom* **1992**, *3*, 398–408.

2. Douglas, D. J. Mechanism of the Collision-Induced Dissociation of Polyatomic Ions Studied by Triple Quadrupole Mass Spectrometry. *J. Phys. Chem.* **1982**, *86*, 185–191.

3. Cooks, R. G., Ed. *Collision Spectroscopy*, 1st ed.; Springer New York, NY, 1978.
